# Genome-wide identification, characterisation and functional evaluation of *WRKY* genes in the sweet potato wild ancestor *Ipomoea trifida* (H.B.K.) G. Don. under abiotic stresses

**DOI:** 10.1186/s12863-019-0789-x

**Published:** 2019-12-03

**Authors:** Yuxia Li, Lei Zhang, Panpan Zhu, Qinghe Cao, Jian Sun, Zongyun Li, Tao Xu

**Affiliations:** 1 Key lab of phylogeny and comparative genomics of the Jiangsu province, Institute of Integrative Plant Biology, School of Life Sciences, Jiangsu Normal University, Xuzhou, 221116 Jiangsu Province China; 2 Department of Plant Biotechnology, College of Agriculture and Life Sciences, Chonnam National University, Gwangju, 500-757 South Korea; 3 Xuzhou Academy of Agricultural Sciences/Sweet Potato Research Institute, CAAS, Xuzhou, 221121 Jiangsu China

**Keywords:** WRKY, Transcription factor, Sweet potato, *Ipomoea trifida*, Abiotic stress

## Abstract

**Background:**

WRKY DNA-binding protein (WRKY) is a large gene family involved in plant responses and adaptation to salt, drought, cold and heat stresses. Sweet potato from the genus *Ipomoea* is a staple food crop, but the *WRKY* genes in *Ipomoea* species remain unknown to date. Hence, we carried out a genome-wide analysis of *WRKYs* in *Ipomoea trifida* (H.B.K.) G. Don.*,* the wild ancestor of sweet potato.

**Results:**

A total of 83 *WRKY* genes encoding 96 proteins were identified in *I. trifida*, and their gene distribution, duplication, structure, phylogeny and expression patterns were studied. *ItfWRKYs* were distributed on 15 chromosomes of *I. trifida*. Gene duplication analysis showed that segmental duplication played an important role in the *WRKY* gene family expansion in *I. trifida.* Gene structure analysis showed that the intron-exon model of the *ItfWRKY* gene was highly conserved. Meanwhile, the ItfWRKYs were divided into five groups (I, IIa + IIb, IIc, IId + IIe and III) on the basis of the phylogenetic analysis on *I. trifida* and *Arabidopsis thaliana WRKY* proteins. In addition, gene expression profiles confirmed by quantitative polymerase chain reaction showed that *ItfWRKY*s were highly up-regulated or down-regulated under salt, drought, cold and heat stress conditions, implying that these genes play important roles in response and adaptation to abiotic stresses*.*

**Conclusions:**

In summary, genome-wide identification, gene structure, phylogeny and expression analysis of *WRKY* gene in *I. trifida* provide basic information for further functional studies of *ItfWRKYs* and for the molecular breeding of sweet potato.

## Background

Due to immobility, plants often encounter challenges from a number of abiotic environments [1]. To adapt to various stress conditions, plants have evolved a series of strategies at multiple levels. At the transcription level, regulating and inducing the temporal and spatial expression of transcription factor (TF) genes are the important approaches to obtain plant stress resistance [2].

The WRKY protein contains a highly conserved 60 amino acid-long WRKY domain at the N-termini and a zinc-finger-like motif (CX_4–5_CX_22–23_ HXH) at the C-termini [3–5]. WRKY TFs are involved not only in biotic stress responses [3, 6, 7], seed coat and trichome development [8–10], embryogenesis [11] and leaf senescence [12, 13] but also in abiotic stress responses and adaptations. In *A. thaliana*, heat treatment inhibits the expression of *AtWRKY33* while induces the expression of *AtWRKY25* and *AtWRKY26* [14]. *VvWRKY24* is induced by cold treatment [15]. Pollen specific expressing gene *AtWRKY34* negatively mediates the cold sensitivity of *Arabidopsis* pollen [16]. Overexpression of *AtWRKY25*, *AtWRKY26*, *AtWRKY39* and *TaWRKY33* can enhance plant resistance to heat stress [14, 17, 18]. Transgenic plants overexpressing *WRKY* genes show increased tolerance to salt and drought stresses, such as overexpressing rice gene *OsWRKY45* and *OsWRKY72* into rice [19, 20], wheat gene *TaWRKY10* into tobacco [21], *Brassica campestris* gene *BcWRKY46* and barley gene *HvWRKY38* into *A. thaliana* [22, 23] and *Gossypium hirsutum* gene *GhWRKY17* in *Nicotiana* [24]. Constitutive expression of corn gene *ZmWRKY23* in *Arabidopsis* also increases the salt tolerance of plants [25]. The *AtWRKY33* and *AtWRKY25* double mutants are sensitive to NaCl, and overexpression of any gene enhances the salt tolerance of *A. thaliana* [14]. In addition, overexpression of *Dendranthema grandiflorum* gene *DgWRKY1* or *DgWRKY3* enhances the salt tolerance of tobacco [21]. Overexpression of *GhWRKY25* increases the salt tolerance but reduces the drought tolerance of *A. thaliana* [26]. BhWRKY1 can bind *BhGolS1* and regulate *BhGolS1* under drought stress [27]. *GmWRKY54* positively regulates the resistance to drought stress in *A. thaliana* [28]. Overexpression of GsWRKY20 reduces the stomatal density and water loss efficiency of *A. thaliana*, thus improving plant drought tolerance [29]. These findings suggest that WRKY TFs are potential targets for improving the abiotic stress resistance of crops. However, the *WRKY* genes in sweet potato remain largely unknown.

Sweet potato (*Ipomoea batatas*) of the family Convolvulaceae is a widely cultivated food crop with excellent agricultural traits. Meanwhile, it is a major forage crop and important bioenergy crop in China. However, the yield and quality of sweet potatoes have been reduced by various environmental pressures [30–35]. Diploid *I. trifida*, as the wild ancestor of cultivated sweet potato [36–38], contains many excellent characteristics, rendering it suitable good model species for the study of sweet potato breeding, construction of a transgenic system and self-incompatibility [39]. In the present study, we performed a genome-wide identification of *I. trifida WRKY* family members*.* Gene duplication, intron/exon distribution and the phylogenetic relationship were analysed. The tissue-specific and stress-responsive expression patterns of *ItfWRKYs* were observed. Moreover, the potential functions of *ItfWRKYs* were predicted and discussed. Our work provides basic information for further functional studies of *ItfWRKYs* and for the molecular breeding of sweet potato in the future.

## Results

### Identification of *ItfWRKYs*

Ninety-six WRKY proteins encoded by 83 *WRKY* genes were identified in *I. trifida*. These *WRKYs* are designated as *ItfWRKY1–ItfWRKY83* according to their positions on the chromosome. Different transcripts from one gene are named similarly. For example, the three transcripts encoded by *ItfWRKY43* are designated as ItfWRKY43.1, ItfWRKY43.2 and ItfWRKY43.3 (Additional file 1: Table S1). As shown in Additional file 2: Fig. S1, all 96 ItfWRKY proteins contained one or two WRKY domains. Although the WRKYGQK domain is highly conserved, glutamine is replaced by a lysine residue in some WRKY proteins (e.g. ItfWRKY1, − 6, − 18, − 31, − 32, − 45 and − 76) (Additional file 2: Fig. S1), which are also found in WRKYs of tomato, *Arabidopsis* and other plant species [3, 40–42]. Similarly, two residues of ItfWRKY36 were replaced, in which glutamine was replaced by threonine residue and lysine was replaced by arginine residue. In addition, most of the ItfWRKY proteins contain C-X4–7-C-X23-H motifs to form C2H2/C2HC-type zinc finger structures (Additional file 2: Fig. S1).

Then, ItfWRKY protein size, protein molecular weight (MW), isoelectric point (pI), phosphorylation site and subcellular location were analysed (Additional file 3: Table S2). Results showed that these 96 ItfWRKYs are 116 (ItfWRKY70) to 697 (ItfWRKY38) aa in length. The MW of the proteins is mostly between 20 and 50 kilodaltons (kDa), and the pIs of these proteins are between 4.92 (ItfWRKY72) and 10.31 (ItfWRKY40). The pIs of about 60% proteins are lower than 7, indicating that most ItfWRKY proteins are acidic under physiological conditions. However, the difference in pI of ItfWRKY proteins under physiological conditions leads to the difference in charge status of groups, causing the binding or dissociation of groups from the proteins and further affecting the protein function. As shown in Additional file 3: Table S2, ItfWRKYs contain 1–10 phosphorylation sites, of which ItfWRKY42, ItfWRKY55 and ItfWRKY75 have at most 10 phosphorylation sites. About 21% of ItfWRKYs contain six or more phosphorylation sites. However, some ItfWRKYs, such as ItfWRKY1, ItfWRKY6 and ItfWRKY31, do not have phosphorylation sites. All ItfWRKYs are predicted to be located in the chloroplast (Additional file 3: Table S2).

### Chromosomal locations and duplication events of the *ItfWRKY* gene family

**Fig. 1:**
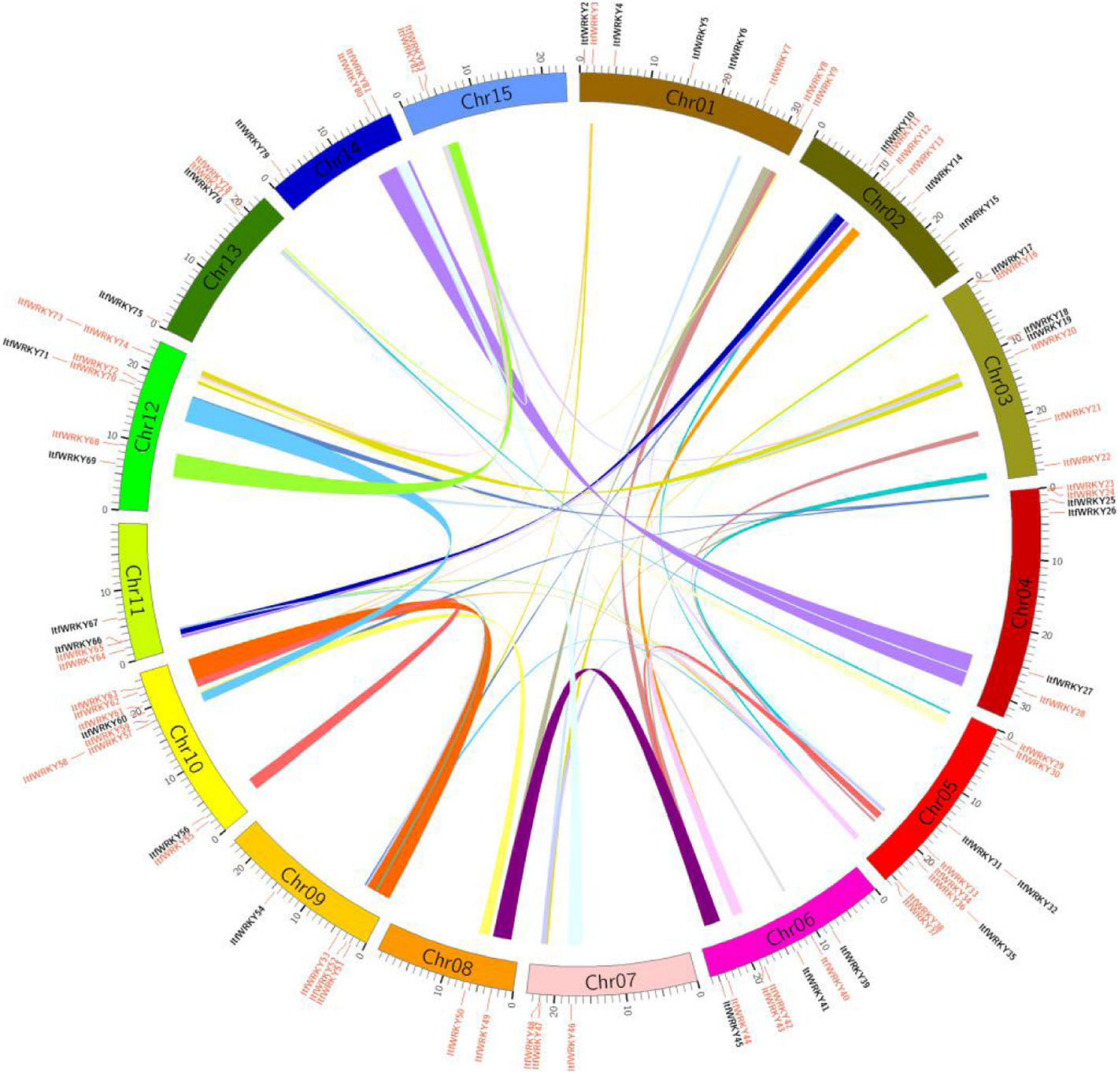
Distribution and segmental duplication of *ItfWRKYs* in *I. trifida* chromosomes. Exactly 82 *ItfWRKYs* were mapped to 15 chromosomes, while one *WRKY (ItfWRKY1)* was located on the unanchored scaffold. Different coloured lines indicate segmental duplication. The red line next to the name indicates the gene cluster on each chromosome. Gene names with collinearity are coloured in red, and no collinear gene names are coloured in black

Among 83 *ItfWRKY* genes, 82 *ItfWRKYs* were mapped on 15 chromosomes, while one *WRKY (ItfWRKY1)* was located on the unanchored scaffold. Figure 1 shows two *ItfWRKY*s on chromosome (Chr) 8 and Chr 15; three on Chr 7 and Chr 14; four on Chr 9, Chr 11 and Chr 13; six on Chr 2 and Chr 4; seven on Chr 3, Chr 6 and Chr 12; eight on Chr 1; nine on Chr 10; and ten *ItfWRKY* genes on Chr 5, indicating that *ItfWRKY*s are distributed unevenly on chromosomes. Segmental and tandem duplication is a main approach for plant gene family expansion [43]. Segmental duplication duplicates multiple genes through polyploidy followed by chromosome rearrangements [43, 44], while tandem duplication is characterised as multiple members of one family occurring within the same intergenic region or in neighbouring intergenic regions (within 200 kb) [45]. We conducted a collinear analysis to study the possible gene duplication types (Fig.1 and Additional file 4: Table S3). Fifty collinear fragments, including *ItfWRKY3–ItfWRKY52*, *ItfWRKY3–ItfWRKY62*, *ItfWRKY7–ItfWRKY47*, *ItfWRKY8–ItfWRKY34* and *ItfWRKY12–ItfWRKY64*, were found using protein-protein BLAST (BLASTP) and Multiple Collinearity Scan tool kit X version (MCSCANX). The above results indicate that segmental duplication played an important role in the *ItfWRKY* family expansion.

### Gene structure analysis of *ItfWRKYs*

**Fig. 2:**
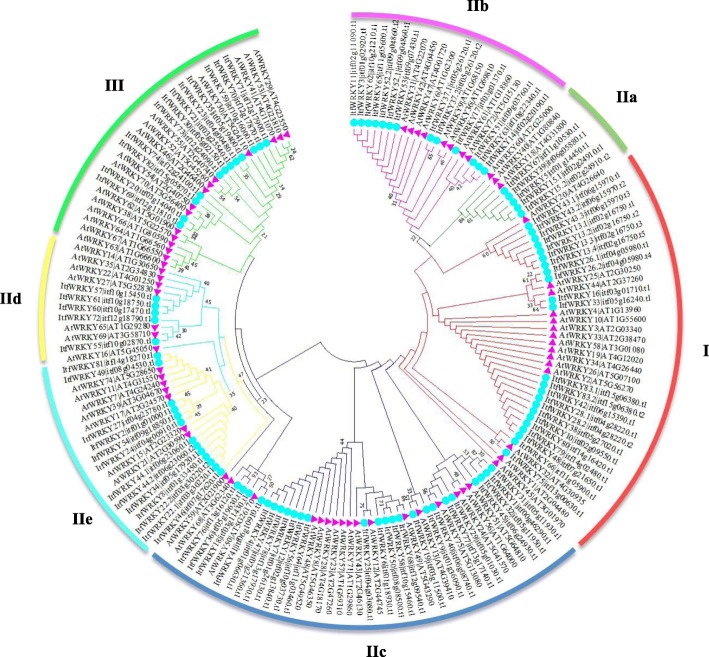
Phylogenetic relationships among the identified WRKY proteins in *Arabidopsis* and *I. trifida*. The 72 *Arabidopsis* and 96 *I. trifida* WRKY protein sequences were used to construct the phylogenetic tree using MEGA7 by the Maximum Likelihood method analysis (1000 replicates). *Arabidopsis* and *I. trifida* genes were indicated at the end of the branches. Subgroups I, IIa, IIb, IIc, IId, IIe and III were named according to *Arabidopsis*. The coloured regions indicate different subfamilies. The blue solid circles and the purple solid triangle indicate the ItfWRKY and AtWRKY proteins, respectively

To identify the structural features of *ItfWRKYs*, we analysed the *ItfWRKY* gene structures. Results showed that the intron number of *ItfWRKY* gene family members ranges from 1 to 7, except *ItfWRKY36*, *ItfWRKY50*, *ItfWRKY58* and *ItfWRKY76* without any intron. Among 96 transcripts, 45 (47%) transcripts contain 2 introns, which occupied the largest percentage. Forty (42%) transcripts contain 3–6 introns. *ItfWRKY48* contains the most introns (7) in the Itf*WRKY* gene family. The diversity in the number of exons and introns leads to a variety of mRNA splicing results during mRNA post-processing, which may be related to protein diversity. As shown in Fig. 2, *ItfWRKYs* were classified into five groups (Cluster I, IIa + IIb, IIc, IId + IIe and III) on the basis of the topology of the Neighbour-joining (NJ) phylogenetic tree (Fig. 2).

### Phylogenetic analysis of ItfWRKY proteins

**Fig. 3:**
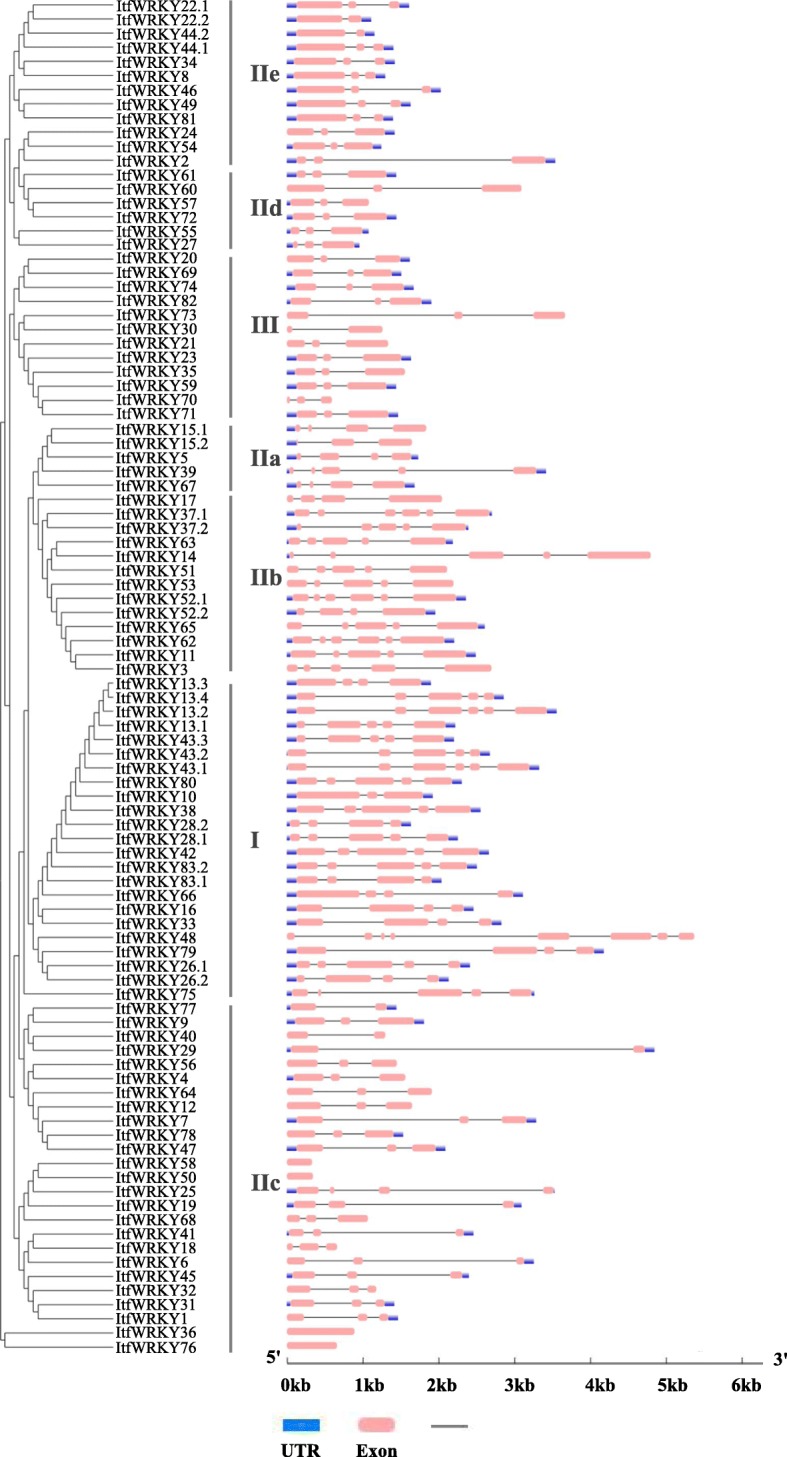
Gene structure of *WRKY* in *I. trifida*. The evolutionary tree of ItfWRKY genes was constructed using MEGA7 and shown on the left. Schematic of exon/intron structure was displayed by the Gene Structure Display Server (http://gsds.cbi.pku.edu.cn/). The exons, introns and UTRs are represented by red solid boxes, black lines and blue boxes, respectively

To study the evolutionary relationship of WRKYs between *I. trifida* and *A. thaliana*, we established a phylogenetic tree of WRKYs. As shown in Fig. 3, the tree contains 72 AtWRKY and 96 ItfWRKY proteins. The *A. thaliana* AtWRKYs were divided into five subgroups (I, IIa + IIb, IIc, IId + IIe and III) [3, 21]. Similar to AtWRKYs, ItfWRKYs were also divided into five subgroups. The ItfWRKYs contain two WRKY domains in Group I, one WRKY domain (with the same Cys2-His2 zinc-finger motif) in Group II and one WRKY domain (with different Cys2-His/Cys Cys2-His2 zinc finger motifs) in Group III. Among these ItfWRKY proteins, 61 in Group II formed the largest branches, of which 5 + 13, 25 and 6 + 12 ItfWRKY proteins were assigned to subgroups IIa + IIb, IIc and IId + IIe, respectively. The second largest group, Group I has 48 WRKYs, including 23 ItfWRKYs and 15 AtWRKYs. Group III has only 27 members (12 ItfWRKYs and 15 AtWRKYs). In addition, the subfamily classification generated from WRKY proteins is consistent with the subgroup classification generated from the intron/exon gene structure analysis.

### Interaction network of the ItfWRKY proteins

**Fig. 4:**
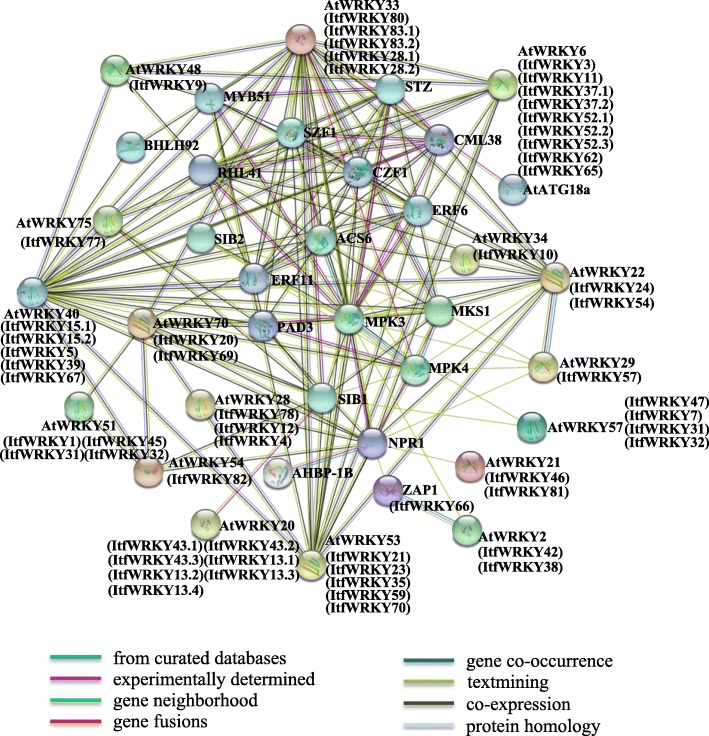
Functional interaction networks of ItfWRKY proteins in *I. trifida* according to orthologues in *A. thaliana*. Network nodes represent proteins, and edges represent protein–protein associations

Understanding the functional relationship of ItfWRKYs is important to understand the regulatory pathway of the family proteins. Therefore, we constructed an ItfWRKY protein interaction network based on *Arabidopsis* homologous genes using STRING software to systematically analyse the interaction of ItfWRKY proteins (Fig. 4). Among the proteins, AtWRKY33 (ItfWRK28.1, − 28.2, − 80, − 83.1 and − 83.2), MPK4, AtWRKY29 (ItfWRKY57), ACS6, AtWRKY22 (ItfWRKY24 and ItfWRKY − 54) and PAD3 are related to the Kyoto Encyclopedia of Genes and Genomes (KEGG) signalling pathway of plant mitogen-activated protein kinases, which include cell defence response, defence response for pathogens, stress adaptation and stress tolerance. WRKY53 (ItfWRK21, − 23, − 35, − 59, − 70 and − 71) is an early factor in drought response, and it regulates stomatal movement and early events of leaf senescence by reducing H_2_O_2_ content and promoting starch metabolism in guard cells [46]. WRKY6 (ItfWRK3, − 11, − 65, − 62, − 52.1, − 52.2, − 52.3, − 37.1 and − 37.2) participates in the control of aging and pathogen defence [47]. WRKY33 (ItfWRKY 28.1, − 28.2, − 80, − 83.1 and − 83.2) specifically interacts with W-box and responds to salt, cold and heat stresses [14, 48]. WRKY28 (ItfWRKY4, − 12 and − 78) and AtbHLH17 confer abiotic stress resistance on *A. thaliana* [49]. These interacting proteins indicate that ItfWRKY proteins have similar functions to *Arabidopsis* proteins. The interaction network of ItfWRKYs provides new research ideas for exploring the new functions of these proteins in the future.

### Tissue-specific expression of *ItfWRKY*s in *I. trifida*

**Fig. 5:**
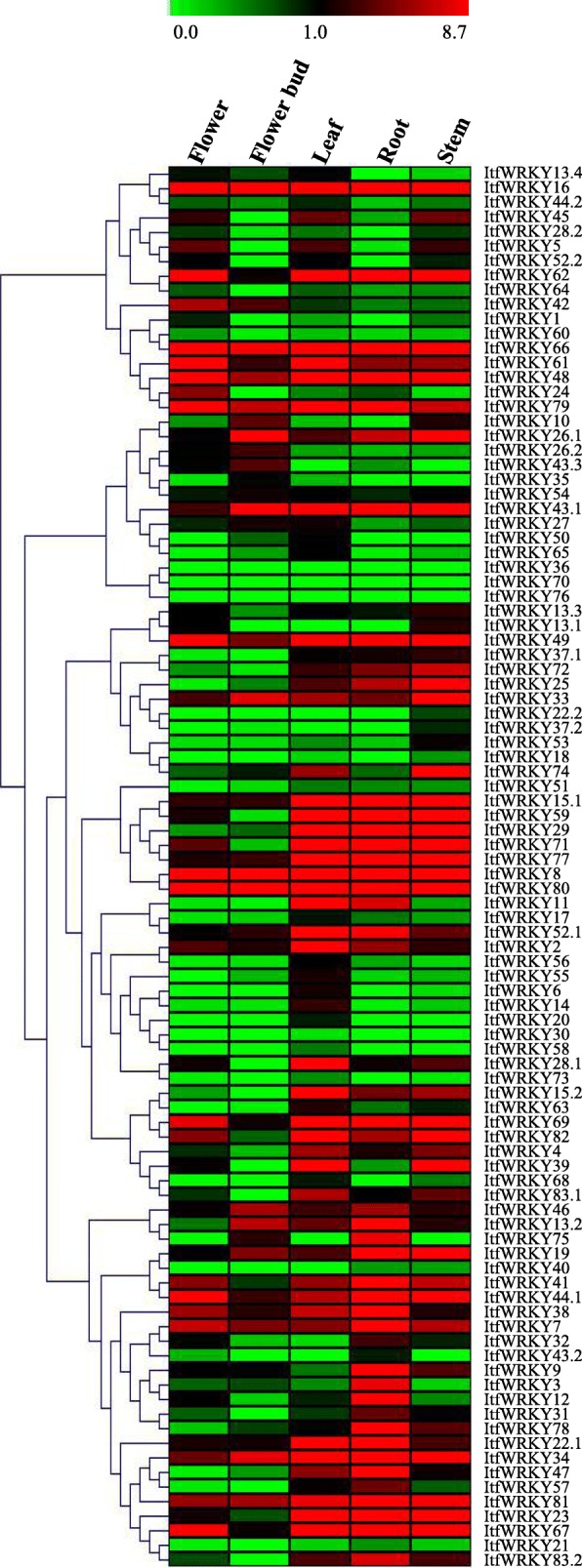
Relative expression levels of *ItfWRKYs* across various tissues. A heat map with clustering is created based on the FPKM value of ItfWRKYs. The coloured scale varies from green to red, indicating relatively low or high expression

**Fig. 6:**
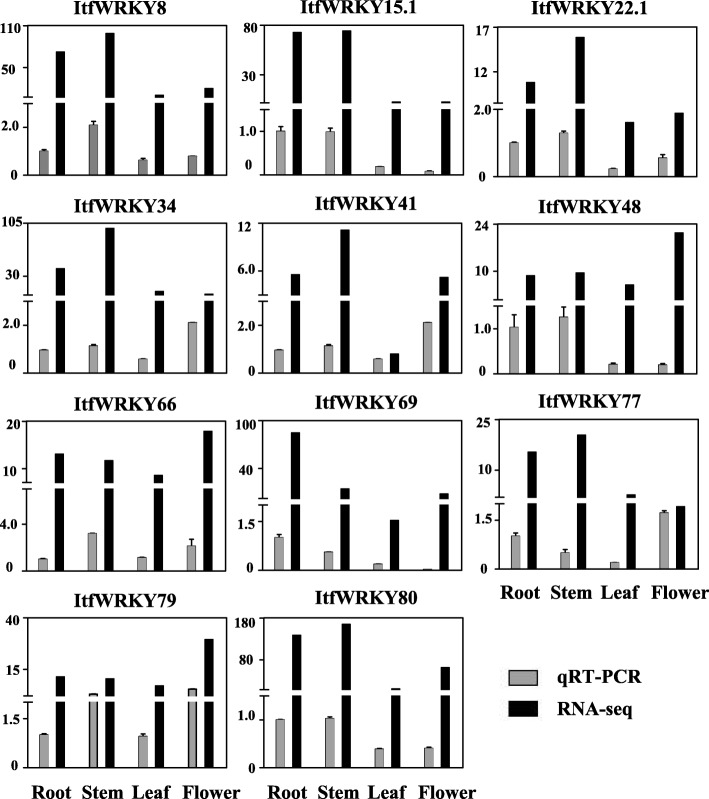
Comparison between quantitative RT-PCR data and RNA-seq data. Relative expression of the 11 selected *ItfWRKYs* was analysed by qRT-PCR. The GAPDH transcript levels were used for normalisation. The y-axis represents the relative expression of the fold. Error bars indicate standard deviation. RNA-seq and qRT-PCR data are represented by black and grey bars, respectively

To evaluate the potential functions of *ItfWRKYs* in plant tissue development, we studied the expression patterns of *ItfWRKY* genes in different tissues (flower, flower bud, root, leaf and stem). As shown in Fig. 5, the expression levels of *ItfWRKYs* varied among different tissues. However, some ItfWRKYs share similar expression patterns. For instance, *ItfWRKY8*, *ItfWRKY16*, *ItfWRKY61*, *ItfWRKY66*, *ItfWRKY48* and ItfWRKY79 were highly expressed in all five tissues, whereas *ItfWRKY1*, *ItfWRKY6*, *ItfWRKY13.4*, *ItfWRKY18*, *ItfWRKY30*, *ItfWRKY35*, *ItfWRKY36*, *ItfWRKY58*, *ItfWRKY68*, *ItfWRKY70* and *ItfWRKY73* were lowly expressed. In addition, different transcripts from the same gene were differently expressed. For example, *ItfWRKY44.1* was highly expressed in flower, leaf, root and stem, whereas *ItfWRKY44.2* was lowly expressed in flower, flower bud, root and stem. Most *ItfWRKY* genes were lowly expressed in flower and flower bud. Interestingly, *ItfWRKY62*, *ItfWRKY67*, *ItfWRKY69* and *ItfWRKY82* were lowly expressed in flower bud but highly expressed in flower. *ItfWRKY3*, *ItfWRKY9*, *ItfWRKY12* and *ItfWRKY78* were expressed highly only in the roots. These results indicate ItWRKYs play diverse roles in plant tissue development. To verify the RNA-seq data of ItfWRKYs, we randomly selected 11 *ItfWRKY* genes and investigated their expression profiles in different tissues (flower, root, stem and leaf) by quantitative polymerase chain reaction (qPCR) (Fig. 6). The gene expression pattern from qPCR results is similar to that from transcriptome sequencing (RNA-seq) data. For instance, the expression levels of *ItfWRKY8*, *ItfWRKY15.1*, *ItfWRKY22.1*, *ItfWRKY48* and *ItfWRKY80* were higher in the roots and stems than in the leaves and flowers (Fig. 6), suggesting that the RNA-seq data are well consistent with the qPCR results.

### Stress-responsive gene expression of *ItfWRKYs* under different abiotic stresses

**Fig. 7:**
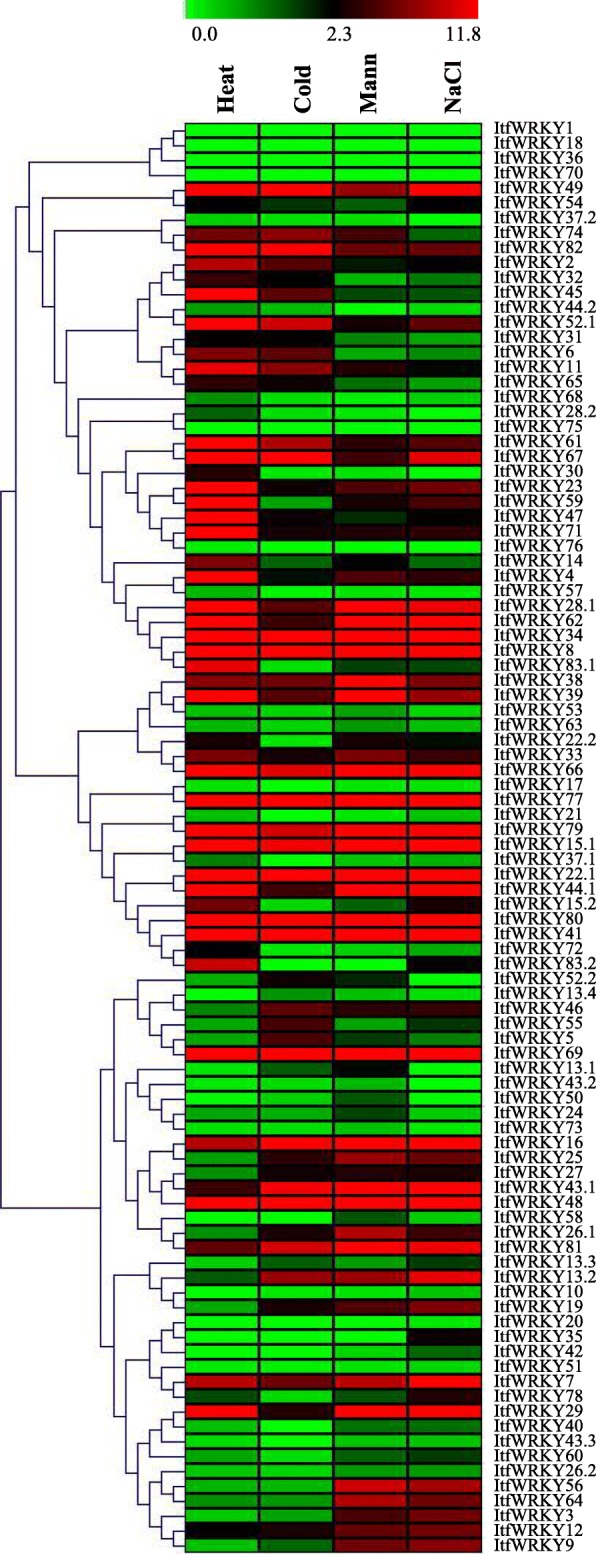
Responses of *ItfWRKYs* to adverse environmental stresses. Expression profiles under four abiotic stresses (cold, heat, drought and salt) were analysed. The coloured scale varies from green to red, which indicates the low or high expression of each gene

WRKYs play key roles in abiotic stress responses in plants [50, 51]. The heat map exhibited the stress-responsive expression patterns of *ItfWRKYs* under salt, drought, cold and heat stresses (Fig. 7). The expression levels of 11 *ItfWRKYs (ItfWRKY8*, *− 15.1*, *− 22.1*, *− 34*, *− 41*, *− 48*, *− 66*, *− 69*, *− 77*, *− 79* and *− 80)* were up-regulated under all four stress conditions, whereas those of 34 *ItfWRKY* genes *(*e.g. *ItfWRKY37.1*, *− 13.1*, *− 43.2*, *− 50*, *− 53*, *− 58* and *− 63.)* were down-regulated. The expression levels of *ItfWRKY7*, *ItfWRKY28.1*, *ItfWRKY29*, *ItfWRKY44.1* and *ItfWRKY62* increased under salt, drought and cold stresses. The expression levels of *ItfWRKY11*, *− 45*, *− 52.1*, *− 61* and *− 82* were induced under cold and heat stresses. Under heat, salt and drought stresses, the expression levels of *ItfWRKY16, ItfWRKY43.1* and *ItfWRKY81* increased. The expression levels of *ItfWRKY2*, *− 4*, *− 14*, *− 23*, *− 47*, *− 59*, *− 71*, *− 83.1* and *− 83.2* were induced by cold stress. The expression levels of *ItfWRKY4*, *− 11*, *− 47*, *− 52.2* and *− 71* were dramatically repressed by salt; those of *ItfWRKY2*, *− 52.1* and *− 67* by drought; those of *ItfWRKY22.2*, *− 28.1*, *− 29*, *− 44.1* and *− 62* by cold; and those of *ItfWRKY13.2*, *− 19*, *− 25* and *− 27* by heat.

**Fig. 8:**
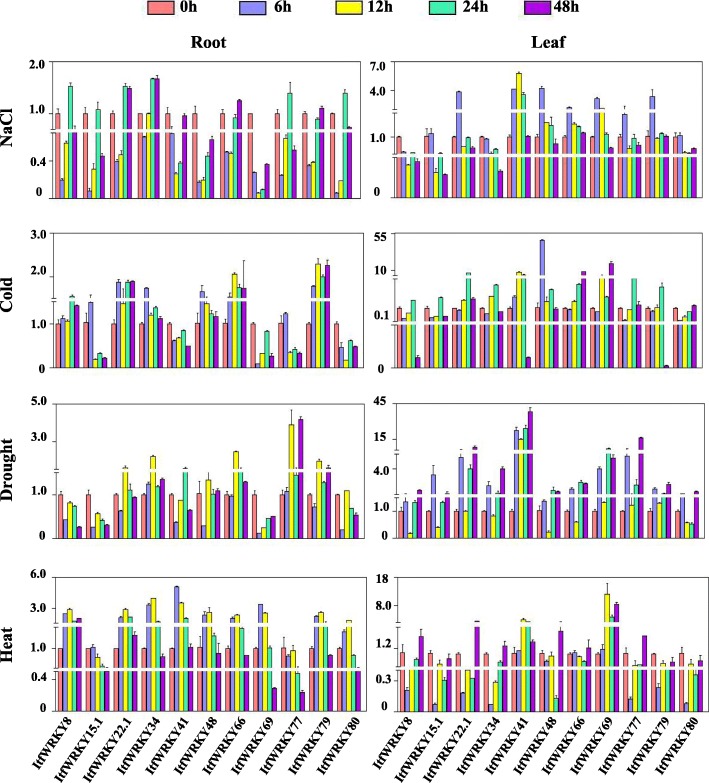
Gene expression confirmed by qRT-PCR under abiotic stresses. The expression at 0 h was set up as 1fold. The y-axis indicates the fold changes of relative gene expression compared with the expression at 0 h. Error bars indicate standard deviation. The expression levels at 0, 6, 12, 24 and 48 h are indicated by red, blue, yellow, green and purple bars, respectively

To further confirm the stress-responsive gene expression of the *ItfWRKYs*, we selected 11 *ItfWRKY* genes (*ItfWRKY-8*, *− 15.1*, *− 22.1*, *− 34*, *− 41*, *− 48*, *− 66*, *− 69*, *− 77*, *− 79* and *− 80*) and checked their expression in the roots and leaves under salt, drought, heat and cold stresses at 0, 6, 12, 24 and 48 h time points (Fig. 8). Results showed that the *ItfWRKY* genes differently responded to various stress treatments. In the roots, the expression levels of *ItfWRKY22.1* and *ItfWRKY34* dramatically increased at 24 and 48 h under salt stress, and those of *ItfWRKY*48 and *ItfWRKY*69 were down-regulated; the expression levels of *ItfWRKY*22.1, − 48, − 66 and − 79 were dramatically up-regulated under cold stress, and that of *ItfWRKY*15.1 was down-regulated; the expression of *ItfWRKY*77 was dramatically up-regulated under drought stress, and those of *ItfWRKY*8, − 15.1 and − 69 were down-regulated. The expression of most *ItfWRKY* genes were up-regulated by heat treatments, except *ItfWRKY15.1* and *ItfWRKY77*. In the leaves, *ItfWRKY41* and *ItfWRKY66* were highly expressed under salt stress; *ItfWRKY22.1*, *− 34*, *− 41*, *− 69* and *− 77* were highly expressed under drought stress; *ItfWRKY66* and *ItfWRKY69* were highly expressed under cold stress; and *ItfWRKY8*, *− 22.1*, *− 41*, *− 48* and *− 69* were highly expressed under heat stress. The up- or down-regulation of the ItfWRKYs suggests that ItfWRKYs play different roles in abiotic stress responses in *I. trifida*.

## Discussion

Sweet potato is a staple crop with important health-care function and industrial value. Since the first WRKY protein *SPF1* was isolated from sweet potato (*I. batatas*) [52], the WRKYs in *Ipomoea* species remain largely unknown. Considering the complicated genetic background of sweet potato due to its hexaploid character, we carried out the genome-wild identification and characterisation of WRKYs in *I. trifida*, the wild ancestor of sweet potato. WRKY as an important TF can rapidly increase its family members to establish a signal transduction network in adversity, which may optimise plant adaptability [53]. Several *WRKY* genes have been identified in plants, such as 72 *WRKY* genes in *A. thaliana* [54], 109 in *Oryza sativa* [20], 197 in *Glycine max* [4], 80 in *Pinus monticola* [55], 35 in *Selaginella involucrate* [56], 45 in *Hordeum vulgare* [41], 38 in *Physcomitrella patens*, 104 in *Populus* spp*.*, 68 in *Sorghum bicolor* and 66 in *Carica papaya* [6]. In the present study, 83 *ItfWRKY* genes encoding 96 transcripts were identified in *I. trifida*.

Segmental duplication of chromosome regions results in a scattered pattern of occurrence, and tandem duplication results in a clustered pattern [57]. Both gene duplication has been observed in the WRKY TF family in *A. thaliana* [58, 59], rice [56, 60, 61], tomato [62] and cucumber [63, 64]. However, tandem gene duplication was not observed in *I. trifida* in our study (Fig. 1), which is similar to the previous report in sesame (*Sesamum indicu*) [65, 66]. A total of 50 segmental duplications was identified in the WRKY gene family in *I. trifida*. In addition, 4 genes *(ItfWRKY35*, *ItfWRKY69*, *ItfWRKY70* and *ItfWRKY71*) in Group III and 6 genes (*ItfWRKY10*, *ItfWRKY26*, *ItfWRKY48*, *ItfWRKY66*, *ItfWRKY75* and *ItfWRKY79*) in Group I do not undergo segmental duplication events. At the same time, ItfWRKYs in Group IIa had no segmental duplication, suggesting that gene duplication did not occur in Group IIa. The above results indicated that segmental duplication is very important for *WRKY* gene family expansion and evolution in *I. trifida*.

In general, intron distributions can be used to study gene evolution, and genes containing many introns are usually considered conservative. The number of *ItfWRKY* introns ranges from 0 to 7. Among them, *ItfWRKY13.2*, *− 37.1*, *− 43.1*, *− 52.1* and *− 62* have 5 introns, and *ItfWRKY48* has 7 introns (Fig. 3), indicating that these ItfWRKYs are highly conserved during evolution. Most *ItfWRKYs* have a highly conserved WRKY (WRKYGQK) domain, whereas individual *ItfWRKYs* show some substitutions, such as glutamine being replaced by lysine residues (ItfWRKY1, − 6, − 18, − 31, − 32, − 45 and 76), glutamine being replaced by threonine residues and lysine being replaced by arginine residues (ItfWRKY36) (Additional file 2: Fig. S1), which is consistent with previous findings [3, 40–42].

To understand the potential function of *ItfWRKYs*, we analysed the tissue-specific expression pattern in the leaves, roots, stems, flowers and flower buds and the stress-responsive expression pattern under four abiotic stresses in *I. trifida*. Most *ItfWRKY* genes are highly expressed in the roots and leaves, whereas a few are expressed in flowers, which is similar to the findings of *ZmWRKY*, *AtWRKY*, *OsWRKY* and *VvWRKY* family members [67]. Thirty-four *ItfWRKYs* were highly expressed in at least three tissues in *I. trifida*. Fifteen of these genes (e.g. *ItfWRKY8*, *ItfWRKY16*, *ItfWRKY48*, *ItfWRKY49*, *ItfWRKY66* and *ItfWRKY80*) are highly expressed in all tissues, suggesting that these highly expressed *WRKY*s are important regulatory factors for tissue development in *I. trifida*. Most of these highly expressed *ItfWRKY*s are in the Group I and II subfamilies. A previous study has shown that Group I *WRKYs*, as the ancestors of other *WRKYs*, are expressed constitutively in different tissues [68]. For example, the Group I *ItfWRKYs* (*ItfWRKY66*, *− 69* and *− 8*0) are expressed in most *I. trifida* tissues and highly expressed under cold, heat and drought stress conditions. By contrast, the expression levels of 12 *ItfWRKY*s (*ItfWRKY18, − 21, − 30, − 36, − 40, − 51, − 58, − 60, − 64, − 70, − 73* and − *76*) were low in all *I. trifida* tissues, and two *ItfWRKY*s (*ItfWRKY30* and *ItfWRKY58*) were only expressed in one tissue. These lowly expressed *ItfWRKYs* are distributed in all *WRKY* subgroups, and most of them are in Groups IIc and IIe. The IIc *WRKY*s in *A. thaliana* (such as *AtWRKY8*, *− 48, − 50* and *− 57*) are involved in pathogen responses and jasmonic acid (JA)- and salicylic acid (SA)-mediated signalling pathways [48]. Thus, we implied that IIc *ItfWRKY*s with low expression levels in most tissues may function in pathogen responses rather than tissue development.

To evaluate the potential functions of ItfWRKYs, we summarised the known functions of WRKY under abiotic stress and compared the gene expression between functional known WRKYs and their *I. trifida* homologs (Table 1)*.* As shown in Table 1, the functions of some *ItfWRKYs* homologous genes have been characterised in *A. thaliana*, *T. aestivum*, *O. sativa*, *G. max*, *G. hirsutum* and other species under various stresses, including cold, salt, heat and drought stresses [14–21, 23–28, 69–75]. For example, overexpression of AtWRKY25 enhances heat and salt tolerance in *Arabidopsis* [14], and its homologous ItfWRKY28.1 is induced by heat and salt stresses, indicating that ItfWRKY28.1 plays the same role under heat and salt stresses. Moreover, similar patterns were investigated for most reported *Arabidopsis WRKYs* and their homologous ItfWRKYs, such as AtWRKY26 and ItfWRKY43.1, AtWRKY33 and ItfWRKY13.3, AtWRKY39 and ItfWRKY44.2, and AtWRKY57 and ItfWRKY4. However, AtWRKY34 is a negative regulator in pollen specific cold response, but the expression of its homologous ItfWRKY38 is up-regulated under cold stress, suggesting that ItfWRKY38 plays a negative role specific in pollen and a positive role in the other tissues under cold stress. For the other homologous ItfWRKYs, most of their gene expression patterns were consistent with those of the functional known WRKYs (Table 1). Taken together, the regulation of WRKYs contributes to its crucial roles in plant abiotic stress responses, which may further establish the complex signalling networks for stress tolerance and adaptation in *I. trifida*.

**Table 1:** Functional evaluation of ItfWRKYs under abiotic stress

Function known WRKYs related abiotic stress				Homologous WRKYs in *I. trifida*		
Species	Protein name	Function	Reference	Protein name	Protein identify (%)	Gene expression under abiotic stress
*Arabidopsis*	AtWRKY25	Overexpression enhanced heat resistance and confer salt tolerance	[14]	ItfWRKY28.1	50.59	Under heat ↑ Under salt ↑
	AtWRKY26	Overexpression enhanced heat resistance	[14]	ItfWRKY43.1	51.69	Under heat ↑
	AtWRKY33	High-temperature represses its expression and induces the expression of *AtWRKY25* and *AtWRKY26*; and confer salt tolerance	[14]	ItfWRKY13.3	52.99	Under heat ↓ Under salt ↑
	AtWRKY34	Negative regulator in pollen specific cold response	[16]	ItfWRKY38	49.34	Under cold ↑
	AtWRKY39	Overexpression enhanced heat stress resistance of transgenic plants	[17]	ItfWRKY44.2	51.30	Under heat ↑
	AtWRKY57	Induced by drought and its expression increase *Arabidopsis* drought tolerance	[18]	ItfWRKY4	84.93	Under drought ↑
*Triticum aestivum*	TaWRKY1	Confer drought and/or heat resistance in *Arabidopsis*	[18]	ItfWRKY65	64.00	Under drought ↓ Under heat ↑
	TaWRKY2	Confer transgenic plants tolerance to drought stress	[69]	ItfWRKY13.1 ItfWRKY13.3	49.49 49.49	Under drought ↓ Under drought ↓
	TaWRKY10	Overexpression enhanced the *tobacco* drought and salt tolerance	[21, 23]	ItfWRKY41	65.17	Under salt ↑ Under drought ↑
	TaWRKY19	Confer transgenic plants tolerance to drought stress	[69]	ItfWRKY79	48.08	Under drought ↑
	TaWRKY33	Confer drought and/or heat resistance in *Arabidopsis*	[18]	ItfWRKY67	41.43	Under drought ↑ Under heat ↑
	TaWRKY44	Confers transgenic tobacco multiple abiotic stress tolerances.	[70]	ItfWRKY79	50.44	Under salt ↑ Under drought ↑ Under heat ↑ Under cold ↑
*Oryza.sativa L*	OsWRKY11	Transgenic lines showed significant heat and drought tolerance	[71]	ItfWRKY64	78.16	Under heat ↓ Under drought ↑
	OsWRKY45	Overexpression enhances salt and drought tolerance	[19, 20]	ItfWRKY70	48.61	Under salt ↓ Under drought ↓
	OsWRKY72	Overexpression enhances salt and drought tolerance	[19, 20]	ItfWRKY77	86.96	Under salt ↑ Under drought ↑
*Glycine max*	GmWRKY13	Transgenic plants increased sensitivity to salt and drought stress while decreased sensitivity to ABA	[28]	ItfWRKY46	63.89	Under salt ↑ Under drought ↑
	GmWRKY21	Confer transgenic plant tolerance to cold stress	[28]	ItfWRKY41	66.22	Under cold ↑
	GmWRKY27	Improved salt and drought tolerance by inhibiting expression of downstream gene GmNAC29	[18, 72]	ItfWRKY15.2	53.74	Under salt ↑ Under drought ↓
	GmWRKY54	Confer transgenic Arabidopsis plants tolerance to salt and drought	[28]	ItfWRKY64	75.61	Under salt ↑ Under drought ↑
*Gossypium hirsutum*	GhWRKY17	Overexpression increases *Nicotiana* tolerance to drought and salt stress	[24]	ItfWRKY30	63.49	Under drought ↓ Under salt ↓
	GhWRKY25	Overexpression plants increased salt tolerance but reduced drought tolerance of *A. thaliana*	[26]	ItfWRKY19	78.57	Under salt ↑ Under drought ↓
	GhWRKY68	Reduces resistance to salt and drought in transgenic *Nicotiana benthamiana*	[75]	ItfWRKY64	75.58	Under salt ↑ Under drought ↑
*Dendranthma grandiflorum*	DgWRKY1	Overexpression enhances salt tolerance	[74]	ItfWRKY77	85.56	Under salt ↑
	DgWRKY3	Overexpression enhances salt tolerance	[74]	ItfWRKY70	65.59	Under salt ↓
*Boea hygrometria*	BhWRKY1	Bind to BhGolS1 to activate the regulation of BhGolS1 under drought stress	[27]	ItfWRKY15.1	56.52	Under drought ↑
*Vitis vinifera*	VvWRKY24	Be induced by cold treatment at all-time points	[15]	ItfWRKY49	76.32	Under cold ↑
*Zea mays*	ZmWRKY23	Enhance tolerance to salt stress	[25]	ItfWRKY72	50.00	Under salt ↓
*Brassica campestris ssp*	BcWRKY46	Overexpression increases *A. thaliana* tolerance to drought and salt stress	[73]	ItfWRKY30	55.74	Under salt ↓ Under drought ↓

↓ indicated decrease of gene expression level; ↑ indicated increase of gene expression level

## Conclusion

In this study, we identified 83 *ItfWRKY* genes encoding 96 WRKY TFs and investigated their gene distribution, structure and evolutionary characteristics. The tissue-specific and stress-responsive expression patterns of *ItfWRKY*s showed that these genes play important roles in plant development, abiotic stress response and adaptation. Our study has established the functional framework of ItfWRKYs, which can facilitate the further functional studies of WRKYs and the molecular breeding of sweet potato.

## Methods

### Data collection and *ItfWRKY* identification

The sequences of *ItfWRKYs* were obtained from the Sweet potato Genomics Resource (http://sweetpotato.plantbiology.msu.edu/), and those of *A. thaliana WRKY* were downloaded from TAIR (https://www.arabidopsis.org/.jsp). The data are shown in Additional file 1: Tables S1. In this study, 114 putative WRKY TFs were retrieved from the sweet potato genome database. The PFAM (http://pfam.xfam.org/) and CDD (http://www.ncbi.nlm.nih.gov/cdd/) databases were used to further confirm whether or not these sequences contain the WRKY domain. Finally, 96 WRKY TFs encoded by 83 *WRKY* genes were identified for this study.

### Chromosomal distribution and gene duplication analysis of *ItfWRKYs*

On the basis of the chromosomal location data provided by the database (http://sweetpotato.Plantbiology.msu.Edu/), the *ItfWRKYs* were mapped on the chromosome of *I. trifida*. Gene duplication were analysed by the Multiple Collinearity Scan toolkit and visualised by Circos (http://circos.ca/) [22, 76].

### Gene structure analysis and motif composition of *ItfWRKY*s

The gene structure of *ItfWRKY* was determined by comparing their genomic sequences with predicted coding sequences using the Gene Structure Display Server (http://gsds.cbi.pku.edu.cn/). The conserved motifs in the ItfWRKY proteins were analysed by the online programmer MEME (http://meme-suite.org/tools/meme).

### Protein properties and phylogenetic tree construction

The molecular weight (MW) and isoelectric points (pIs) of the ItfWRKY proteins were determined using the ProtParam program (ExPASy tools) (http://expasy.org/tools/). Meanwhile, phosphorylation analysis and subcellular localisation prediction were carried out using the P3DB online tool (http://www.p3db.org/) and the software WoLFPSORT (https://wolfpsort.hgc.jp/) forecast, respectively. The neighbour-joining (NJ) phylogenetic trees of *I. trifida* and *A. thaliana* WRKY proteins were generated using MEGA7 [77]. In addition, a protein network of functional interactions between *I. trifida* and *A. thaliana* was constructed by STRING software (https://string-db.org/cgi/network.pl).

### Transcriptome and quantitative real-time polymerase chain reaction (PCR) analysis

The *ItfWRKY* RNA-seq data (in Additional file 5: Table S4 and Additional file 6: Table S5) were downloaded from the sweet potato database (http://sweetpotato.plantbiology.msu.edu/). The gene expression levels of *ItfWRKYs* were calculated as fragments per kilobase of exon per million fragments mapped (FPKM). The heat maps of *ItfWRKY* expression profiles were generated using Mev.4.9.0. Total RNA was isolated by using the RNAprep Pure Kit (Tiangen Biotechnology, Beijing, China). The first chain of DNA was synthesised by PrimeScript™ RT Reagent Kit (Tsingke, Nanjing, China) and used as the template for quantitative PCR. The primers used for PCR are listed in Additional file 7: Table S6, and qPCR was carried out on ABI Step One Plus instrument (Biosoar, Nanjing, China).

### Plant growth and stress treatments

*I. trifida* plants were collected from Xuzhou Academy of Agricultural Science. The plants were grown as previously described [78]. In brief, vermiculite, perlite and soil were mixed at a ratio of 1:1:4 and placed under 16 h light/8 h darkness and 26 °C growth temperature. To study the tissue-specific patterns of *ItfWRKYs*, we collected the roots, leaves and stems of four-week-old *I. trifida* seedlings and flowers of two-month-old plants [39]. For the analysis of stress-responsive expression patterns, four-week-old plants were divided into five treatment groups: control group, salinity treatment group (200 mM NaCl solution), cold treatment group (12 °C), drought treatment group (300 mM mannitol solution) and heat treatment group (40 °C). Leaves and roots were sampled at 0, 6, 12, 24 and 48 h.

## Supplementary information

**Additional file 1: Table S1.** Accession numbers of WRKY genes in Ipomoea trifida and Arabidopsis thaliana.

**Additional file 2: Fig S1.** Alignment of ItfWRKY domain sequences. The alignment was performed by the Multiple interface page (http://multalin.toulouse.inra.fr/multalin/). The conserved WRKY aa and zinc-finger motifs are highlighted in red. Gaps are indicated by dashes.

**Additional file 3: Table S2.** Informations of ItfWRKYs.

**Additional file 4: Table S3.** Chromosomal locations and segmental duplication of ItfWRKY genes.

**Additional file 5: Table S4.** Relative expression levels of ItfWRKYs in various tissues.

**Additional file 6: Table S5.** Expression pattern of ItfWRKYs under abiotic stresses.

**Additional file 7: Table S6.** Primers of the ItfWRKY genes and housekeeping gene for qRT-PCR.

## Data Availability

All data generated or analysed during this study are included in this published article and its additional files.

## Abbreviations

BLASTP: Protein-protein BLAST
Chr: Chromosome
JA: Jasmonic acid
kDa: Kilodalton
KEGG: Kyoto Encyclopedia of Genes and Genomes
MCSCANX: Multiple Collinearity Scan tool kit X version
NJ: Neighbour-joining
pI: Isoelectric point
Protein MW: Protein molecular weigh
qPCR: quantitative polymerase chain reaction
RNA-seq: Transcriptome sequencing
SA: Salicylic acid
TF: Transcription factor
WRKY: WRKY DNA-binding protein
