# Supplementary figures and images for: Genome-wide identification, characterisation and functional evaluation of WRKY genes in the sweet potato wild ancestor Ipomoea trifida (H.B.K.) G. Don. under abiotic stresses
Source: BMC Genet. 2019 Dec 3;20:90. doi: 10.1186/s12863-019-0789-x (PMC6889533; doi:10.1186/s12863-019-0789-x)

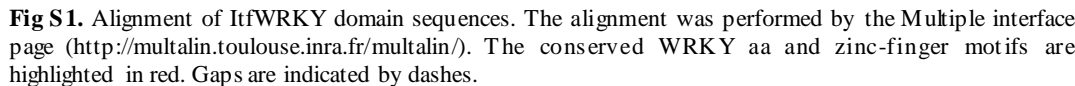

Supplement: Supplementary file 2 — Additional file 2: Fig S1. Alignment of ItfWRKY domain sequences. The alignment was performed by the Multiple interface page (http://multalin.toulouse.inra.fr/multalin/). The conserved WRKY aa and zinc-finger motifs are highlighted in red. Gaps are indicated by dashes. [file 12863_2019_789_MOESM2_ESM.pdf]
